# Metabolic modulation of chromatin: implications for DNA repair and genomic integrity

**DOI:** 10.3389/fgene.2013.00182

**Published:** 2013-09-17

**Authors:** Jinping Liu, Jeongkyu Kim, Philipp Oberdoerffer

**Affiliations:** Laboratory of Receptor Biology and Gene Expression, National Cancer Institute, National Institutes of HealthBethesda, MD, USA

**Keywords:** chromatin, DNA repair, metabolism, aging, cancer

## Abstract

The maintenance of genomic integrity in response to DNA damage is tightly linked to controlled changes in the damage-proximal chromatin environment. Many of the chromatin modifying enzymes involved in DNA repair depend on metabolic intermediates as cofactors, suggesting that changes in cellular metabolism can have direct consequences for repair efficiency and ultimately, genome stability. Here, we discuss how metabolites may contribute to DNA double-strand break repair, and how alterations in cellular metabolism associated with both aging and tumorigenesis may affect the integrity of our genomes.

## INTRODUCTION

Dynamic chromatin reorganization affects numerous cellular processes, including differentiation and development. In recent years, it has become apparent that chromatin does not only determine gene expression and epigenetic integrity of nuclear DNA, but directly contributes to the repair of genomic lesions, of which DNA double-strand breaks (DSBs) are the most toxic (Lukas et al., 2011; Shi and Oberdoerffer, 2012; Soria et al., 2012; Smeenk and van Attikum, 2013). The need for chromatin reorganization during eukaryotic DNA repair becomes apparent in light of the tight packaging of DNA in nuclear space: in humans and mice, approximately every 200 bp, 146–147 bp of DNA are wrapped around a nucleosome, which forms the structural core of the chromatin fiber. Each nucleosome consists of a histone octamer containing two molecules of H2A, H2B, H3, and H4, or variants thereof, assembled in one H3–H4 heterotetramer and two H2A–H2B heterodimers. Nucleosomes are subject to further condensation via the H1 linker histones, which contact the exit/entry points of the DNA strand on the nucleosome and facilitate higher order chromatin folding (Luger et al., 1997; Hansen, 2002). DNA lesions that occur in the context of chromatin must, thus, be made accessible to the repair machinery, a process that involves remodeling and/or depletion of nucleosomes as well as an array of epigenetic modifications, which can affect both DNA and histone proteins (Polo and Jackson, 2011; Smeenk and van Attikum, 2013). While the most prevalent epigenetic alterations of DNA are methyl-based modifications of cytosines in CpG context, histones are subject to a wide range of reversible, post-transcriptional modifications including acetylation, methylation, phosphorylation, ubiquitylation, sumoylation, and ADP ribosylation (Kouzarides, 2007). Over the past decade, all of these modifications have been implicated in the modulation of DSB repair (Lukas et al., 2011; Shi and Oberdoerffer, 2012; Soria et al., 2012; Smeenk and van Attikum, 2013).

Many of the enzymes responsible for the deposition or removal of chromatin modifications require metabolic intermediates as cofactors or substrates for their activity. Changes in metabolism and, consequently, metabolite availability are tightly associated with both aging and cancer (Ward and Thompson, 2012; Cosentino and Mostoslavsky, 2013), raising the intriguing possibility that age- or disease-associated metabolic alterations can result in the deregulation of DNA repair by altering chromatin function. In the following, we will highlight the impact of metabolites on chromatin modifiers involved in DSB repair and speculate how changes in metabolite abundance may influence genome maintenance and nuclear integrity.

## METABOLITES AS MEDIATORS OF CHROMATIN CHANGES

Metabolic programs coordinate energy intake and its use to control cell survival and growth. Eukaryotic cells rely on oxidative metabolism as the most efficient way to produce energy from nutrients. The mitochondrial tricarboxyclic acid (TCA) cycle oxidizes pyruvate derived from glucose or fatty acids to CO_2_, thereby generating adenosine triphosphate (ATP) and the reduced form of the redox cofactors nicotinamide adenine dinucleotide (NADH) and flavin adenine dinucleotide (FADH_2_). Oxidation of NADH and FADH_2_ to NAD^+^ and FAD, in turn, promotes the production of ATP via oxidative phosphorylation and the electron transport chain (ETC). ATP and NADH can also be generated from glucose directly via glycolysis. For a detailed description of these metabolic pathways, we refer the reader to a comprehensive review by Locasale and Cantley (2011). In addition to energy production, cellular metabolism ensures the balanced generation of intermediate metabolites, which are required for cell growth and/or serve as co-enzymes or substrates for a number of enzymatic processes (Yuan et al., 2013). The latter is particularly relevant during conditions of nutrient excess, when cells were found to switch to what is known as aerobic glycolysis to maintain growth. The same metabolic switch is also observed in many cancer cells, where it was first described by Otto Warburg and is, thus, referred to as Warburg effect (Warburg, 1956; Ward and Thompson, 2012).

**FIGURE 1:**
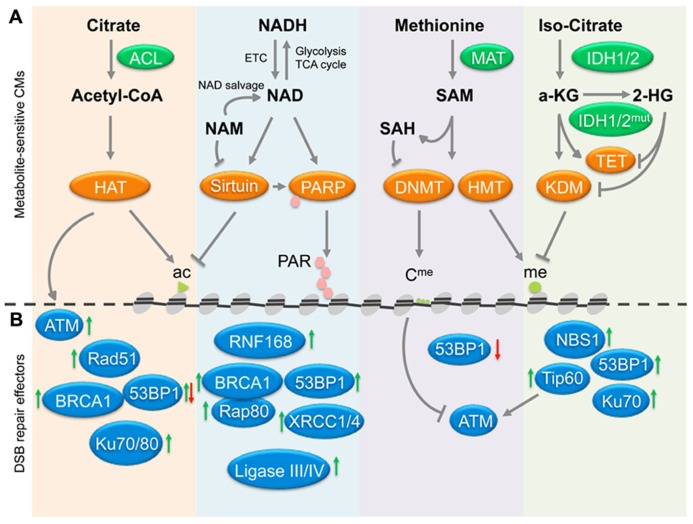
**Cross-talk between metabolites, chromatin and DSB repair. (A)** The deposition of chromatin marks is influenced by metabolites, which function as cofactors or substrates for the indicated chromatin modifiers (CMs). Central pathways affecting histone acetylation, deacetylation, PARylation, and histone/DNA methylation and demethylation are shown, arrows indicate positive regulation/deposition of chromatin marks, blunted arrows depict negative regulation/removal of chromatin marks. **(B)** Chromatin marks deposited or removed through pathways in **(A)** have been implicated in the modulation of the recruitment of the indicated repair factors, suggesting a possible link between metabolites and DSB repair. Green arrows indicate that a given chromatin modification results in increased recruitment/activation of the indicated repair factors, red arrows depict impaired activation/recruitment in the presence of the same modification. See **Table 1** for a detailed list of modifiers and modifications involved in DSB repair. In addition to chromatin, the HAT Tip60 can acetylate and activate the central DDR mediator ATM kinase (gray arrows), whereas ATM activity is negatively affected by DNA methylation (blunted gray arrow). Ac, acetylated histone; me, methylated histone; C^me^, methy-cytosine; PAR, poly-(ADP-ribose); ETC, electron transport chain; HAT, histone acetyl transfrease; HMT, histone methyltransferase; KDM, lysine demethylase. See text for enzyme and metabolite abbreviations.

Accumulating evidence suggests that metabolic changes associated with age and/or tumor development may not simply be a consequence of the latter. This notion has been discussed in a number of number of recent reviews, which implicate altered metabolite profiles both in the regulation of p53 activity (Vousden and Ryan, 2009) and the epigenetic control of gene expression in aging and disease (Vousden and Ryan, 2009; Ward and Thompson, 2012; Yun et al., 2012; Cosentino and Mostoslavsky, 2013). Here, we propose that metabolite-associated changes in chromatin may further contribute to aging and malignant transformation through the deregulation of DNA repair. We focus on chromatin modifications that have been extensively investigated in the context of DNA DSB repair and highlight the key metabolite cofactors and/or substrates required for these modifications: specifically we discuss (i) acetyl-CoA-dependent acetylation and NAD^+^-dependent deacetylation of histones, (ii) NAD^+^-dependent poly-ADP ribosylation, and (iii) *S*-adenosyl-L-methionine (SAM)-dependent methylation as well as FAD/α-ketoglutarate-dependent demethylation of histones and/or DNA (see **Figure 1A**).

### HISTONE (DE)ACETYLATION

The acetylation of histones increases the negative charge of chromatin fibers, which renders chromatin structurally accessible and is, therefore, generally associated with less densely packed chromatin (euchromatin) and concomitant active gene transcription (Kouzarides, 2007). Acetylation of a specific histone residue (H3K56) has further been linked to the (re-)assembly of nucleosomes following replication or DNA repair (Downs, 2008; Das et al., 2009), and a similar process has been described for H3K4 acetylation during the re-assembly of densely packed, heterochromatic DNA (Xhemalce and Kouzarides, 2010). Histone acetylation requires the acetyl-group donor acetyl-CoA. In mammalian cells, nuclear acetyl-CoA is generated from glucose that has been converted to citrate via the TCA cycle. Unlike acetyl-CoA, citrate can cross the mitochondrial membrane and serves as substrate for ATP-citrate lyase (ACL), which is localized in the cytoplasm and nucleus and is essential for non-mitochondrial acetyl-CoA production (Bauer et al., 2005). Changes in glucose availability or ACL levels are, thus, expected to have a direct impact on acetyl-CoA-dependent nuclear acetylation reactions. Indeed, Thompson and colleagues demonstrated that depletion of ACL results in reduced histone acetylation and concomitant gene silencing, whereas growth factor signaling as well as increased glucose metabolism resulted in excess acetyl-CoA production, increased acetyl-histone levels and increased gene expression (Wellen et al., 2009).

Like the acetylation of histones, their deacetylation can be modulated by metabolite availability. Perhaps the most prominent example are sirtuins, or class III histone deacetylases (HDACs), which depend on the oxidized form of NADH, NAD^+^, as a co-factor. Both overall NAD^+^ abundance and the cellular ratio of NAD^+^/NADH are, therefore, critical determinants of sirtuin activity (Imai et al., 2000; Lin et al., 2002). Notably, sirtuin-dependent deacetylation converts its co-factor NAD^+^ to nicotinamide (NAM), a vitamin B3 precursor that functions as a non-competitive sirtuin inhibitor (Bitterman et al., 2002). NAM can be recycled to NAD^+^ via NAD salvage pathways, and the enzymes involved in this pathway were found to promote sirtuin activity in yeast and mammalian cells (Anderson et al., 2003; Yang et al., 2007). Although sirtuin function has been linked to numerous cellular processes, their impact on chromatin reorganization is emphasized by reports demonstrating sirtuin-dependent gene repression as well as maintenance of repressive chromatin structures, including telomeric and centromeric DNA (Vaquero et al., 2004, 2007; Michishita et al., 2008, 2009; Oberdoerffer et al., 2008; Yang et al., 2009; Haigis and Sinclair, 2010; Palacios et al., 2010).

Taken together, these findings indicate that both nutrient excess and aberrant cell growth may favor an overall increase in histone acetylation and active transcription, by increasing acetyl-CoA production and simultaneously inhibiting NAD^+^-dependent histone deacetylation.

### POLY-(ADP-RIBOSYL)ATION

In addition to sirtuins, NAD^+^ is also a co-factor for poly-(ADP-ribose) polymerases (PARPs), a family of enzymes that plays an important role in the regulation of chromatin packaging, transcription, DNA replication, and DNA repair. PARPs utilize NAD^+^ to transfer ADP-ribose to histones, non-histone proteins, and PARP itself. The poly-(ADP-ribosyl)ation (PARylation) of non-histone proteins has been implicated in numerous cellular processes ranging from transcription to intracellular trafficking to cell division and has been comprehensively discussed elsewhere (Schreiber et al., 2006; Beneke, 2012). Perhaps most relevant for this review, PARP proteins have been directly linked to chromatin (re)organization in response to a variety of developmental or environmental cues, such as steroids, heat shock, or genotoxic stress. PARP activation results in the evolutionarily conserved, PAR-dependent stripping of chromatin proteins from DNA, thereby promoting chromatin opening and concomitant transcriptional activation (Tulin and Spradling, 2003; Martinez-Zamudio and Ha, 2012). Moreover, PARP1 promotes the exclusion of the linker histone H1 at transcription start sites and is required to maintain promoter-associated active chromatin marks (Krishnakumar and Kraus, 2010). Conversely, inhibition of PARP activity was found to cause an increase in the number and density of heterochromatin foci as well as DNA methylation (de Capoa et al., 1999), which may at least in part be due to PAR-mediated inhibition of DNA methyltransferase 1 (DNMT1; Reale et al., 2005). Like most post-translational modifications, PARylation is a reversible process. The hydrolysis of PAR chains is catalyzed by poly-ADP ribose glycohydrolase, which does not require additional metabolite cofactors and will, thus, not further be discussed (Feng and Koh, 2013).

### HISTONE AND DNA (DE)METHYLATION

In contrast to acetylation and PARylation, both DNA and histone methylation are frequently associated with the establishment and/or maintenance of repressive chromatin domains. Densely packed chromatin, or heterochromatin, which is largely inaccessible to the transcription machinery, is highly CpG methylated and harbors characteristic methyl-histone marks, such as H3K9me3 (constitutive heterochromatin) or H3K27me3 (facultative heterochromatin). Notably, histone methylation can also be associated with active transcription, as is the case for promoter associated H3K4 trimethylation and co-transcriptionally deposited H3K36 trimethylation. For a comprehensive overview of the occurrence and function of DNA and histone methylation, we refer the reader to recent reviews (Cedar and Bergman, 2009; Black et al., 2012).

Both DNA and histone methyltransferase enzymes (DNMTs and HMTs) rely on SAM as a common co-substrate for methyl group transfers. SAM is generated by adding ATP to methionine via methionine adenosyltransferase (MAT). As a result of the methyl transfer reaction, SAM is converted to *S*-adenosyl homocysteine (SAH), which is a potent inhibitor of both HMT and DNMT activity. SAH can be cleared by hydrolysis to adenosine and homocysteine. Methylation of DNA and histones is thus sensitive to cellular SAH, SAM, and homocysteine levels (reviewed in Grillo and Colombatto, 2008; Yun et al., 2012). Notably, in addition to being restored to methionine and eventually SAM, homocysteine can serve as a precursor for glutathione (GSH) synthesis. The latter serves as a major cellular redox buffer in response to oxidative stress, which in turn is tightly linked to both cancer and aging (Haigis and Yankner, 2010). Depletion of GSH due to increased reactive oxygen species (ROS) can divert homocysteine away from the methionine recycling pathway, which may, in turn, result in a decrease in SAM and inhibition of methyltransferase activity (Yun et al., 2012).

Although methylation was long considered to be an irreversible epigenetic mark, several enzymes that allow for the conversion and/or removal of methyl groups from DNA and histones have been uncovered in recent years. The first demethylase to be discovered was LSD1, a lysine demethylase, which specifically removes methyl groups from mono- and dimethylated H3K4 (Shi et al., 2004). The LSD1-mediated demethylation reaction requires FAD as a cofactor, which is reduced to FADH2 during the amine oxidation of N-methylated lysine substrates, providing a link between energy consumption and LSD1 activity (Shi et al., 2004; Forneris et al., 2005).

In addition to LSD1, a second, larger family of Jumonji C (JmjC) domain containing histone demethylases has been identified, which harbors α-ketoglutarate (α-KG)-dependent dioxygenase activity (Black et al., 2012). α-KG is a TCA cycle intermediate that is generated from isocitrate by isocitrate dehydrogenases (IDH) 1 (cytosolic) and 2 (mitochondrial; Ward et al., 2010). Notably, α-KG levels do not only affect histone demethylases, but also the recently discovered TET family of 5-methylcytosine hydroxylases, which are involved in the demethylation of DNA (Kriaucionis and Heintz, 2009; Tahiliani et al., 2009; Ward and Thompson, 2012). The function of both TET and JmjC enzymes is directly affected by several recurrent somatic mutations of IDH1 and IDH2, which are commonly found in glioma (75%; Parsons et al., 2008) and acute myeloid leukemia (20%; Mardis et al., 2009). These mutations result in aberrant enzyme activity, which converts α-KG to 2-hydroxy-ketoglutarate (2-HG; Yang et al., 2012). 2-HG is a competitive inhibitor of α-KG-dependent enzymatic reactions (Xu et al., 2011), which in turn leads to genome-wide alterations in both histone and DNA methylation (Xu et al., 2011; Lu et al., 2012).

Together, these observations underline the power of metabolic changes with regard to epigenetic maintenance, be it in response to altered growth conditions and/or nutrient availability, or due to mutations in key metabolic enzymes. In the following we will discuss the possibility that altered metabolite profiles may, at least in part, contribute to age-related genome instability and malignant transformation by altering the regulation of DNA repair through chromatin.

## METABOLITE-SENSITIVE ASPECTS OF CHROMATIN-DIRECTED DSB REPAIR

Double-strand breaks are incompatible with DNA replication and thus represent one of the most toxic DNA lesions. To eliminate DSBs, eukaryotic cells generally employ one of two repair pathways: non-homologous end-joining (NHEJ) or homologous recombination (HR). HR allows for the error-free repair of a DSB in the presence of a homologous, undamaged DNA template, usually the sister chromatid, and is typically restricted to S and G2 phases of the cell cycle. To allow for invasion of the undamaged template, the recombination event requires the generation of single-stranded DNA (ssDNA) via the resection of broken DNA ends. NHEJ, on the other hand, involves the religation of the broken ends with no or minimal resection of DNA ends and can occur in all phases of the cell cycle. In case of minimal end resection, the lack of template, however, bears the risk of mutations upon repair (see Ciccia and Elledge, 2010; Polo and Jackson, 2011; Chapman et al., 2012) for recent, comprehensive reviews).

Over the past decade, it has become evident that chromatin modifications play a critical role in most if not all phases of NHEJ and HR. A common theme to both repair pathways is the need to make chromatin accessible for repair factors (Lukas et al., 2011; Soria et al., 2012; Smeenk and van Attikum, 2013). This process involves the remodeling, depletion, and re-assembly of nucleosomes, the acetylation of histones as well as the incorporation of histone variants associated with active chromatin, such as H2AZ (Murr et al., 2006; Xu et al., 2012; Smeenk and van Attikum, 2013). In addition, numerous histone modifications have been shown to function as assembly platforms for repair factors through direct interaction of these proteins with the modified histone. Perhaps the most prominent such modification is the phosphorylation of H2AX on serine 139 (referred to as γ-H2AX), which is mediated by PI3 kinase like linase (PIKK) family members ATM, ATR, or DNA-PKcs. γ-H2AX provides a high affinity binding site for MDC1, which in turn orchestrates the recruitment of a variety of functionally distinct effector proteins, such as 53BP1 and BRCA1, two central mediators of NHEJ and HR, respectively (Bekker-Jensen and Mailand, 2010). In addition to histone modifications common to most if not all repair pathways, such as γ-H2AX, recent evidence has identified a growing list of more selective modifications, which can help fine-tune or even determine the choice of repair factors, and thus the repair pathways involved in the resolution of the break (reviewed in Lukas et al., 2011; Shi and Oberdoerffer, 2012; Soria et al., 2012; Smeenk and van Attikum, 2013). In the following we will focus on the metabolite-sensitive chromatin modifications described above and discuss their impact on DSB repair and genomic integrity (see **Figure 1B** and **Table 1**).

**Table 1:** Metabolite-sensitive chromatin modifiers involved in DSB repair.

Metabolite	Modification	Enzyme	Histone/protein target	Effect on DSB repair
Acetyl-CoA	Acetylation	Tip60	H4K16ac ↑	53BP1 ↑/↓
				BRCA1 ↑
				Rad51 ↑
				ATM ↑
		GCN5	H3K9ac ↑ H3K56ac ↑	Chromatin relaxation?
			Chromatin re-assembly
		CBP/P300	H3K56ac ↑	Chromatin re-assembly
				Ku70/80 ↑
		HAT1	H4K5/K12ac ↑	Chromatin re-assembly
				Rad51 ↑
NAD^+^	Deacetylation	SIRT1	H3K56ac ↓	Chromatin re-assembly
			H4K16ac ↓	53BP1 ↑?
		SIRT2	H3K56ac ↓	Chromatin re-assembly
		SIRT6	H3K9ac ↓	Chromatin condensation?
	PARylation	PARP	unknown	XRCC1/LigaseIII ↑
				53BP1 ↑
				BRCA1/RAP80 ↑
				RNF168 ↑
				SIRT6 ↑
				XRCC4/ligase IV
		SIRT6	PARP1	PARP1 ↑
SAM	Methylation	SETD8	H4K20me2 ↑	53BP1 ↑
		SUV420	H4K20me2 ↑	53BP1 ↑
		DOT1L	H3K9me2 ↑	53BP1 ↑
		SUV39H1/2	H3K9me3 ↑	Tip60/ATM ↑
		MLL	H3K4me3 ↑	S-phase checkpoint ↑
		SETMAR	H3K36me2 ↑	NBS1 ↑
				Ku70 ↑
		EZH2	H3K27me3 ↑	Chromatin condensation?
		MMSET	H4K20me ↑	53BP1 ↑
FAD	Demethylation	SPR-5	H3K4me2 ↓	DSB repair in meiosis
α-KG/2-HG	Demethylation	JHDM1A	H3K36me2 ↑ (α-KG)/↓ (2-HG)	Tip 60 ↑/↓?
		JMJD2A	H3K9/K36me2/3 ↑/↓	Tip60 ↑/↓
		JMJD2B	H3K9me2/3 ↑/↓	γ-H2AX turnover
		JMJD2D	H3K9/K36me2/3 ↑/↓	Tip60 ↑/↓

###  ACETYL-CoA AND DSB-ASSOCIATED HISTONE ACETYLATION

Acetyl-CoA-dependent histone acetylation is a central aspect of the DNA damage response, as it facilitates the relaxation of DSB-surrounding chromatin and concomitant repair factor access. Perhaps the most prominent DSB repair-associated histone acetyltransferase (HAT) is Tip60 (or KAT5). Tip60 is rapidly recruited to sites of DNA damage in a manner that is dependent on the break-sensing Mre11–Rad50–Nbs1 (MRN) complex, and promotes acetylation of DSB-surrounding histone H4 (Murr et al., 2006) as well as the DDR mediator ATM (Sun et al., 2005). The latter was found to amplify ATM activity and is essential for the DSB-induced, ATM-dependent checkpoint response (Sun et al., 2007). Similarly, Tip60-mediated acetylation of histone H4 was found to promote the recruitment of several repair factors central to both HR and NHEJ, including BRCA1, Rad51, and 53BP1 (Murr et al., 2006), suggesting a synergistic effect of Tip60-induced ATM and H4 acetylation. Notably, recent evidence suggests that H4K16 acetylation by Tip60 can selectively interfere with the recruitment of 53BP1, thereby promoting BRCA1 recruitment (Hsiao and Mizzen, 2013; Tang et al., 2013). Moreover, depletion of both Tip60 and its co-factor TRRAP have been shown to inhibit DNA repair by HR, although decreased histone acetylation was also shown to impair NHEJ (Murr et al., 2006; Hsiao and Mizzen, 2013; Tang et al., 2013), suggesting a complex and likely dynamic role for acetylation and deacetylation in the regulation of DNA repair that warrants further investigation.

Several additional HATs have been linked to DSB-associated chromatin remodeling over the past years. GCN5 (or KAT2A) links the dynamic acetylation and deacetylation of several residues on H3 and H4 to HR in yeast (Tamburini and Tyler, 2005). Notably, induction of DSBs in mammalian cells has been linked to a transient reduction in H3K9ac as well as H3K56ac, and GCN5 was identified as a HAT responsible for re-establishing the latter mark (Tjeertes et al., 2009). In addition to GCN5, CREB-binding protein (CBP)/P300 has been shown to promote the acetylation of H3K56 in response to DNA damage or replication stress. Histones bearing acetylated K56 are, in turn, (re-)assembled into chromatin in yeast, flies, and human cells, forming foci that colocalize with sites of DNA repair (Das et al., 2009). Depletion of CBP/P300 reduced recruitment of the end-joining factors Ku70/80 and, consequently, NHEJ efficiency, supporting a functional link between nucleosome turnover and repair by NHEJ (Ogiwara et al., 2011). Similar to the role of H3K56ac, HAT1-mediated K5/K12 acetylation of newly synthesized H4 is required for their efficient incorporation into DSB-proximal chromatin. However, HAT1 depletion has been linked to defects in HR rather than NHEJ due to impaired recruitment of the ssDNA binding protein Rad51 (Yang et al., 2013). Interestingly, in addition to and likely preceding HAT-mediated chromatin re-assembly, increased acetylation of several of the core histones was found to promote their polyubiquitin-independent degradation in response to DNA damage. The resulting chromatin disassembly is thought to facilitate repair factor access and involves the proteasome activator PA200, which binds to acetylated histones (Qian et al., 2013). Notably, both DNA damage and aging have recently been shown to promote histone loss, suggesting a mechanistic link between the two processes, possibly through a common increase in chromatin decondensation (Feser et al., 2010; O’Sullivan et al., 2010). Increased histone acetylation, in turn, was found to activate ATM in the absence of DNA breaks, indicative of a positive feedback that may eventually result in deregulated DDR, genomic instability, and/or cell cycle arrest observed with age (Bakkenist and Kastan, 2003; Kaidi and Jackson, 2013).

Together, these findings demonstrate that the modulation of histone acetylation has a significant, yet complex impact on the DDR and can affect repair via both NHEJ and HR. It will be of interest to determine how changes in the essential HAT co-factor acetyl-CoA, and by extension glucose availability, affect DSB repair and genomic stability in the context of either excessive growth (tumorigenesis) or growth arrest (senescence).

### NAD^+^-DEPENDENT HISTONE DEACETYLATION AND PARylation IN DSB REPAIR

Double-strand break-associated histone acetylation is a dynamic process that is counteracted by HDACs, which can be sensitive to metabolite availability as is the case for NAD^+^-dependent sirtuins [see Histone (de)acetylation]. To date, at least three of the four nuclear mammalian sirtuins, SIRT1, SIRT2, and SIRT6 have been implicated in DNA damage control (Haigis and Sinclair, 2010). However, although these proteins have been reported to affect both HR and NHEJ by deacetylating non-histone DNA repair proteins, evidence for sirtuin-mediated histone deacetylation at sites of DSBs is largely circumstantial. Both SIRT1 and SIRT6 are recruited to sites of DNA damage (Oberdoerffer et al., 2008; Kaidi et al., 2010; Toiber et al., 2013) and SIRT6 was recently found to mediate DSB-specific H3K56 deacetylation (Toiber et al., 2013). Notably, recruitment of SIRT6 to DSB-flanking chromatin may help activate its deacetylase activity, as the latter was found to require interaction with intact nucleosomes (Gil et al., 2013). In addition to H3K56ac deacetylation, SIRT6 promotes the recruitment of the SNF2H remodeling factor and concomitant chromatin accessibility at sites of damage. If these processes are functionally linked remains to be determined (Toiber et al., 2013). Similar to SIRT6, SIRT1 and SIRT2 are able to deacetylate H3K56ac, which was found to modulate CBP/P300-dependent H3K56 acetylation and concomitant chromatin re-assembly at DNA breaks (Das et al., 2009). In addition, SIRT1 was shown to deacetylate H4K16ac, which may in turn counteract Tip60 activity and, thus, alter ATM activation as well as the recruitment of DSB repair factors (Vaquero et al., 2004; Tang et al., 2013). Finally, SIRT6 and to a lesser extent SIRT1 can deacetylate H3K9ac, which may have implications for the modulation of DSB-induced chromatin accessibility (Vaquero et al., 2004). However, evidence for sirtuin-dependent, DSB-specific deacetylation of many of these marks is missing. Future work is expected to provide a better understanding of how sirtuins may modulate the DDR via targeted histone deacetylation.

While the role of sirtuins in DSB-associated chromatin reorganization is only emerging, the NAD^+^-dependent PARP enzymes PARP1 and PARP2 are well-established modulators of DSB-proximal chromatin accessibility and, consequently, DSB repair. PARP activation is one of the first events in the DDR, which results in extensive PARylation in the vicinity of DSBs and facilitates the recruitment of early repair sensors such as Mre11 (Schreiber et al., 2006; Yelamos et al., 2011). Deletion of PARP1 and PARP2 results in increased DNA damage sensitivity, increased genomic instability and, in case of PARP1, increased tumor formation (Menissier de Murcia et al., 2003; Tong et al., 2007). PARP1 has been found to interact with the NHEJ factors XRCC1 and ligase III and is required for NHEJ under conditions that involve minimal end resection (known as alternative NHEJ; Audebert et al., 2004). Notably, the latter is inhibited by 53BP1, suggesting that PARP1 may counteract 53BP1 recruitment (Bouwman et al., 2010; Bunting et al., 2010; Chapman et al., 2012). Consistent with this notion, PARP1 was recently shown to promote HR by regulating chromatin expansion and spatial accumulation of the BRCA1/RAP18 complex at DSBs (Smeenk et al., 2013). Interestingly, the DDR-relevant target(s) for PARylation remain to be identified, and although PARylation of histone tails has been reported (Messner et al., 2010), it is unclear whether these modifications affect DSB repair. Notably, SIRT6 was recently found to alter DSB repair by mono-ribosylating and, thus, activating PARP1. SIRT6-dependent activation of PARP is dependent on oxidative stress, suggesting that NAD^+^-dependent repair factors may help integrate DNA repair and stress signaling pathways (Mao et al., 2011).

Together, these findings indicate that, similar to acetyl-CoA, alterations in cellular NAD^+^ levels can modulate DSB repair. Consistent with the opposing activities of HATs and HDACs in the DDR, acetyl-CoA is associated with nutrient excess, whereas NAD^+^ is elevated under conditions of nutrient deprivation. Notably, PARylation and histone deacetylation appear to have similarly opposing effects on chromatin packaging, yet both PARPs and sirtuins depend on NAD^+^. It will, thus, be of particular interest to determine if and how PARylation and sirtuin-mediated histone deacetylation cooperate in a scenario where NAD^+^ is limiting, as observed both with age and in highly glycolytic tumor cells (Elstrom et al., 2004; Braidy et al., 2011; Massudi et al., 2012). Interestingly, PARP can negatively regulate SIRT1 expression, underlining the functional crosstalk between the two enzymatic processes (Bai et al., 2011a, b). Consistent with an overall genome-protective role for NAD^+^, high levels of NAD^+^ were recently reported to correlate with radio-protection in human glioma cells (Sahm et al., 2013), whereas low level NAD^+^ were associated with increased DSB damage in aged cells (Braidy et al., 2011).

### SAM AND DSB-ASSOCIATED HISTONE METHYLATION

Like PARylation and histone acetylation, their methylation is emerging to be an integral aspect of the cellular response to DSBs. And like all acetylation reactions are dependent on acetyl-CoA, all methyl-group additions require a single intermediate metabolite, SAM, as essential co-factor, making both processes intricately linked to nutrient availability and cell growth. However, while acetylation is often thought of as a means to change DSB-proximal chromatin structure and accessibility, methylation of histones appears to be a more selective modulator of repair factor recruitment (van Attikum and Gasser, 2009; Bekker-Jensen and Mailand, 2010; Shi and Oberdoerffer, 2012). Perhaps the most prominent example for the latter is the NHEJ-associated repair factor 53BP1, the recruitment of which to DSBs depends on the binding of a subset of dimethylated histone lysine residues via its conserved tandem tudor domains (Huyen et al., 2004; Botuyan et al., 2006). Both 53BP1 and its yeast ortholog Crb2 show strong affinity for dimethylated H4K20 compared with unmethylated or trimethylated H4K20 (Botuyan et al., 2006). H4K20 methylation appears to be established prior to DNA damage and involves the HMTs SETD8 and SUV420 (Hsiao and Mizzen, 2013), although DSB-induced H4K20 dimethylation via MMSET has also been reported (Pei et al., 2011). In addition to H4K20, H3K79 was found to interact with 53BP1 when dimethylated by DOT1L, albeit with weaker affinity (Huyen et al., 2004; Botuyan et al., 2006). Underlining the requirement for dimethyl-histone binding, deletion of the HMTs responsible for either H4K20me2 or H3K79me2 resulted in impaired formation of 53BP1 foci in response to DNA damage and concomitant DNA repair defects (Huyen et al., 2004; Wakeman et al., 2012; Hsiao and Mizzen, 2013). Similar to H4K20me2, H3K79me2 appears to be established prior to DNA damage rather than being induced by the DDR (Huyen et al., 2004), raising the intriguing question of how pre-existing, DNA damage-independent histone marks can elicit a DSB-specific damage response. This conundrum was addressed recently, when Durocher and colleagues demonstrated that 53BP1 recruitment requires the ubiquitination of H2A-K15 as a DSB-induced, additional histone modification for optimal binding to H4K20me2 containing nucleosomes. H2A-K15 ubiquitination is dependent on RNF168, an E3 ligase that is recruited to chromatin following DSB induction (Fradet-Turcotte et al., 2013). Notably, 53BP1 binding to H4K20 dimethylated nucleosomes can also be negatively regulated via acetylation of K16 on the same H4 tail (Tang et al., 2013).

Evidence for the involvement of additional, pre-existing histone methyl marks in the DDR is rapidly accumulating. Both H3K9 and H3K36 trimethylation were shown to activate the HAT activity of DSB-associated Tip60 (Sun et al., 2009). Indeed, the direct interaction of Tip60 with H3K9me3 at DSBs was found to be essential for Tip60-mediated activation of ATM and the downstream DDR effectors. Consequently, deletion of the enzymes responsible for H3K9 trimethylation, SUV39H1, and SUV39H2, abolished Tip60 HAT activity in response to DSB induction (Sun et al., 2009). DSB-induced structural changes in break-proximal chromatin have been implicated in the exposure of these trimethylated histone marks, and remarkably, experimentally induced chromatin perturbation can elicit activation of Tip60/ATM without the induction of DSBs (Bakkenist and Kastan, 2003; Kaidi and Jackson, 2013).

In addition to pre-existing methyl marks, a number of DSB-induced changes in histone methylation have now been linked to DSB repair: the mixed-lineage leukemia histone methyltransferase MLL was found to activate the S-phase checkpoint in response to DSBs via trimethylation of H3K4 (Liu et al., 2010), although the mechanistic basis for this phenomenon remains to be investigated. SETMAR/Metnase was found to mediate dimethylation of H3K36 at DSBs, which appears to promote NHEJ via the recruitment of Nijmegen breakage syndrome 1 (NBS1) and Ku70 to DSB sites via a yet to be determined pathway (Fnu et al., 2011). Finally, the polycomb-associated H3K27 HMT EZH2 is recruited to DSBs, coinciding with rapid but transient H3K27 trimethylation. Notably, this process appears to be independent of PIKK signaling but requires PARP activity. Although its functional implications for the DDR remain to be investigated, depletion of EZH2 renders cells sensitive to ionizing radiation (IR; Chou et al., 2010).

Together, these findings suggest that changes in both steady state and DSB-induced histone methylation can affect the induction and/or execution of the DDR, which is in turn directly affected by SAM availability. Notably, the latter is negatively affected by oxidative stress, which competes for the SAM-precursor homocysteine due to GSH depletion [see Histone and DNA (De)methylation]. Oxidative stress is frequently observed in both aged and tumor cells, suggesting that histone methylation-dependent aspects of the DDR may be impaired under these conditions.

### FAD-, α-KG-, AND DSB-ASSOCIATED HISTONE DEMETHYLATION

Since the discovery of the first histone demethylase, LSD1, demethylases have been identified for many of the known methylated histone residues (Black et al., 2012). LSD1 and the closely related LSD2 are unique in that they required FAD as a cofactor, whereas JmjC domain containing dioxygenases, the largest class of histone lysine demethylases, require α-KG. Given this distinction, LSD1 and JmjC demethylases have the potential to respond differently to nutrient availability, which in turn may have consequences for the metabolic regulation of DSB repair.

The *C. elegans* LSD1 otholog SPR-5, which demethylates H3K4me2 was shown to mediate DSB repair in meiosis (Nottke et al., 2011). However, a direct role for LSD1/LSD2 in the DDR in mammalian cells remains to be identified. In contrast, several JmjC-type demethylases have been directly linked to DSB repair. JHDM1A (KDM2A) mediated demethylation of H3K36me2 at DSBs was found to counteract SETMAR-mediated H3K36 dimethylation, and concomitantly, repair via NHEJ (Fnu et al., 2011). Recently, JMJD2B (KDM4B) was found to promote demethylation of H3K9me2 and H3K9me3 in response to DNA damage and KDM4B depletion resulted in accelerated resolution of DNA breaks as indicated by reduced frequency of γ-H2AX foci late in the DDR (Young et al., 2013). In addition, the closely related KDM4 demethylases JMJD2A (KDM4A) and JMJD2D (KDM4D), which mediate demethylation of trimethylated H3K9/H3K36 or H3K9, respectively (Klose et al., 2006; Whetstine et al., 2006), were shown to interfere with the activation of Tip60 by reducing its binding to DSB-proximal chromatin (Sun et al., 2009). Notably, KDM4A can bind to methylated H4K20 and is degraded following DNA damage, thereby allowing for the accumulation of 53BP1 at DSBs. It is, however, unclear if this process involves demethylation of methyl-H4K20 (Mallette et al., 2012).

Together, these findings suggest that the negative regulation of DNA damage signaling is a common feature of KDM function in DNA repair, whereas inhibition of KDM activity may result in increased DDR activation. It is, therefore, tempting to speculate that the JmjC-type KDM inhibitor 2-HG, which is the product of a tumor-associated gain of function mutation in IDH genes [see Histone and DNA (De)methylation], may alter repair efficiency in tumor cells. Overexpression of IDH mutant enzymes as well as administration of 2-HG have been shown to promote an increase in several histone methyl marks associated with DSB repair, including H3K9me3, H3K27me3, and H3K4me3 and, to a lesser extent H3K36me3 and H3K79me2 (Lu et al., 2012). Increased H3K9me3 abundance may activate Tip60, and consequently ATM-mediated damage signaling. Similarly, increased H3K79 methylation may promote the recruitment of 53BP1. Notably, the latter has been shown to interfere with HR in the absence of BRCA1, thereby accounting for genomic instability observed in BRCA1 mutant tumors (Bouwman et al., 2010; Bunting et al., 2010). Together, these findings suggest that interference with histone demethylation may contribute to aberrant DDR activation and/or altered repair outcome, which may eventually contribute to genomic instability and, thus be exploited for genotoxic cancer therapy.

### DNA METHYLATION AND DEMETHYLATION AT DSBs

Like the methylation of a variety of histone residues, DNA methylation was found to accumulate at sites of DSBs (Cuozzo et al., 2007). This process appears to require the maintenance methyltransferase DNMT1, which is rapidly and transiently recruited to DSB. DNMT1 recruitment is dependent on its ability to interact with both the DNA polymerase processivity factor PCNA and ATR, suggesting a role in DSB repair during DNA replication in S phase. Notably, reduced DNMT1 activity results in aberrant activation of the DDR in the absence of damage (Ha et al., 2011). Consistent with DNMT1 being a suppressor of abnormal DDR activation, deletion of DNMT1 in cancer cells was found to result in cell cycle arrest in G2/M and mitotic catastrophy in escapes (Chen et al., 2007). Like DNMT1, the DNMT1-associated protein 1 (DMAP1) was found to link DSB-associated DNA methylation to DSB repair. DMAP1 is selectively enriched in DSB-flanking chromatin and DMAP1 depletion can cause (persisting) hypomethylation, suggesting that DMAP1 activates DNMT1 preferentially at sites of DNA damage. Notably, DMAP1 depletion resulted in enhanced HR, which further supports a repressive role for methylation in this process (Lee et al., 2010). DMAP1 was also found to associate with the TIP60/P400 HAT complex, and appears to be required for Tip60-mediated H4K16 acetylation and concomitant ATM activation. Consistent with the latter, DMAP1 depletion caused increased IR sensitivity and a decrease in 53BP1 foci formation (Murr et al., 2006; Penicud and Behrens, 2013). It will be interesting to determine if DMAP1-dependent ATM activation involves DNA methylation via DNMT1. Given the inhibitory effect on HR and 53BP1 recruitment, it is further tempting to speculate that DNA methylation and DMAP1 can serve as a modulator of repair pathway choice.

In contrast to DNA methylation, evidence for DSB-associated DNA demethylation is missing. Perhaps to most promising candidates for the latter are the TET family of proteins, however, a role for TET proteins in DSB repair remains to be identified. Notably, both methylation of DSB-proximal DNA and its putative demethylation are dependent on metabolic intermediates, suggesting, that the effect of altered SAM, 2-KG, and 2-HG levels may not be limited to histone methylation, but affect the modulation of DSB repair via DNA methylation as well.

## PERSPECTIVE

The repair of DNA breaks is central to cell survival, genome integrity and proper cell function. The repair machinery is, however, sensitive to perturbations in cellular homeostasis, resulting in increased DNA damage accumulation and defective genome maintenance in both aged and transformed cells. Here, we propose that metabolic changes associated with either of these processes may be a causal contributor to genomic instability due to defective chromatin-directed DSB repair (see **Figure 1** and **Table 1**). Although the link between metabolic changes and altered chromatin organization has been established with regard to the regulation of gene expression, its link to DNA damage control remains to be identified. Based on the observations discussed here, we believe that the latter is not a matter of “if,” but “how,” although many questions remain to be answered. One of the most intriguing aspects of the involvement of metabolites in DSB repair is the potential to modulate repair efficiency by changing nutrient availability, cellular metabolism, and/or metabolite abundance. This may eventually be utilized to promote DNA repair and, thus, prevent damage accumulation and genomic aberrations observed with age. On the other hand, targeted metabolic changes may be employed to impair DSB repair, thus enhancing genotoxic therapy in cancer treatments. However, due to the many facets of chromatin in the regulation of DSB repair, we are still far from being able to predict how even a defined metabolic change may affect repair outcome. Investigating the interplay between metabolism, chromatin, and repair, is thus vital to further our understanding of how aging and tumorigenesis affect our genomes.

## Conflict of Interest Statement

The authors declare that the research was conducted in the absence of any commercial or financial relationships that could be construed as a potential conflict of interest.
